# Regulation of Mitochondrial Dynamics by Aerobic Exercise in Cardiovascular Diseases

**DOI:** 10.3389/fcvm.2021.788505

**Published:** 2022-01-13

**Authors:** Changping Gu, Jie Yan, Liang Zhao, Guanghan Wu, Yue-lan Wang

**Affiliations:** ^1^Department of Anesthesiology and Perioperative Medicine, The First Affiliated Hospital of Shandong First Medical University, Taian, China; ^2^Shandong Provincial Qianfoshan Hospital, Shandong Institute of Anesthesia and Respiratory Critical Medicine, Jinan, China; ^3^Department of Anesthesiology and Perioperative Medicine, Shandong Provincial Qianfoshan Hospital, Shandong University, Jinan, China

**Keywords:** aerobic exercise, mitochondrial dynamics, myocardial mitochondria, cardiovascular disease, mitochondrial fusion, mitochondrial fission

## Abstract

Mitochondrial dynamics, including continuous biogenesis, fusion, fission, and autophagy, are crucial to maintain mitochondrial integrity, distribution, size, and function, and play an important role in cardiovascular homeostasis. Cardiovascular health improves with aerobic exercise, a well-recognized non-pharmaceutical intervention for both healthy and ill individuals that reduces overall cardiovascular disease (CVD) mortality. Increasing evidence shows that aerobic exercise can effectively regulate the coordinated circulation of mitochondrial dynamics, thus inhibiting CVD development. This review aims to illustrate the benefits of aerobic exercise in prevention and treatment of cardiovascular disease by modulating mitochondrial function.

## Introduction

Cardiovascular diseases (CVDs) are the leading cause of global mortality and a major contributor to disability worldwide (1). CVD pathogenesis is a complex biological process, but few feasible targets exist to prevent or reverse CVD (2). The American College of Cardiology recently reported significant progress in drug treatment for CVD; however, these drugs always have negative long-term effects, causing a reduction of 10–20% in the left ventricular mass (3). Therefore, it is essential to find effective non-pharmaceutical therapies. Aerobic exercise has many benefits to the cardiovascular system, such as improving the mechanical properties of the heart and enhancing its contractility to reduce the incidence of many CVDs. Some of these improvements may arise from effects on mitochondria.

Mitochondria are the energy factories of cells, producing adenosine triphosphate (ATP), and are the main source of cellular reactive oxygen species (ROS). Dysfunctional mitochondria limit energy production, increase ROS production, and transmit apoptotic signals, leading to tissue damage and organ dysfunction (4). Mitochondrial dynamics, which include mitochondrial biogenesis, fusion, fission, and autophagy, play an important role in maintaining mitochondrial homeostasis and ensuring mitochondrial function (5). Mitochondrial dynamics are particularly important for cells with high energy requirements, such as cardiomyocytes, which continuously require ATP to support heart function (6). Therefore, regulating mitochondrial dynamics through effective interventions is crucial for preventing and treating CVDs (7). In animal models of heart failure, Campos et al. found that aerobic exercise improved the mitochondrial fusion/fission balance and restored cardiac autophagy flux, thereby improving cardiac function (8). Here, we will address the biological mechanisms of mitochondrial dynamics in CVDs and discuss the mechanism of aerobic exercise in improving cardiovascular diseases, thereby providing valuable clues for CVD prevention and treatment.

## Mitochondrial Dynamics and CVDs

**Figure 1:**
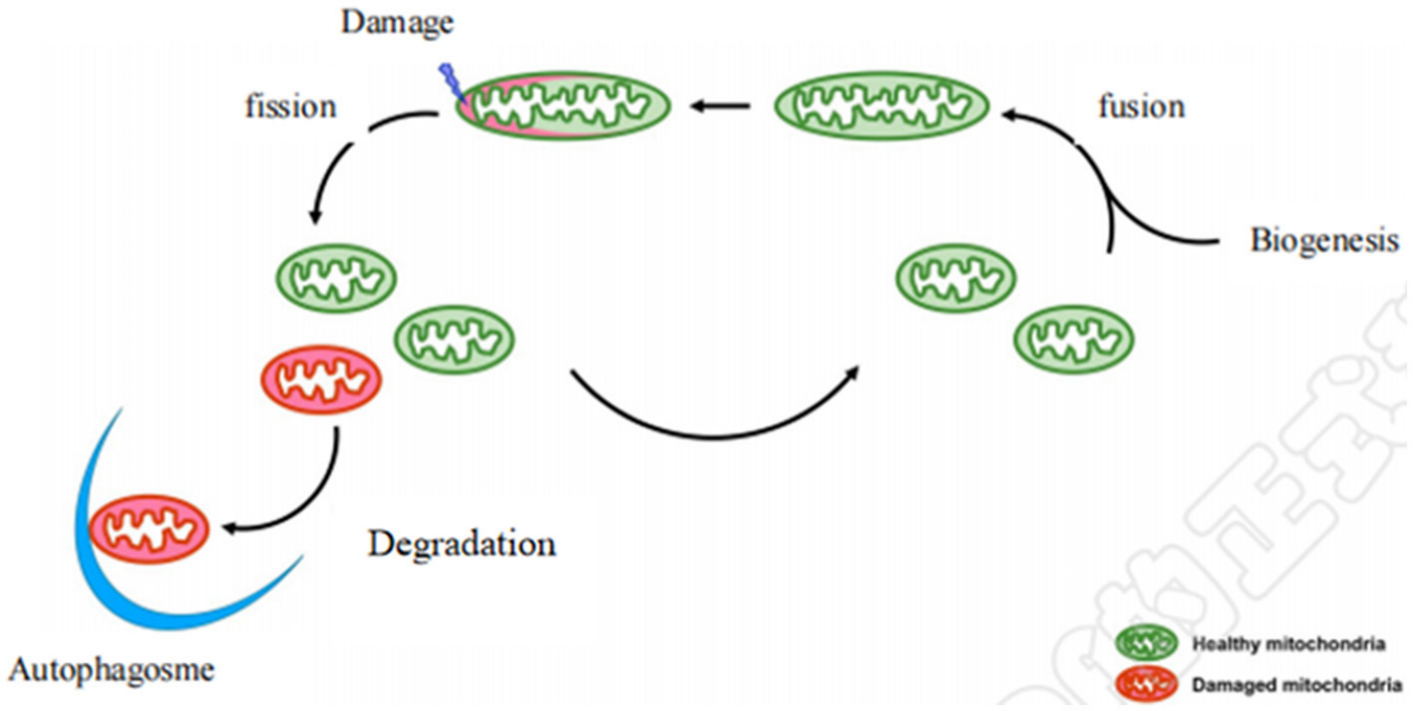
Diagram of mitochondrial dynamics. Mitochondrial biogenesis is the process by which new mitochondria are formed in the cell after mitochondrial fusion. Mitochondrial biogenesis is activated by numerous different signals which can lead to mitochondrial fission and damaged mitochondria are degraded by autophagy.

Mitochondria are highly dynamic organelles, constantly undergoing a coordinated cycle of biogenesis, fusion, fission, and degradation (Figure 1) (9), and forming a complex network to respond to dynamic changes in energy requirements (10). Maintaining healthy and functional mitochondrial networks is critical for physiological adaptation and coping with stress during development and throughout life (11). Mitochondrial dynamics consist mainly of mitochondrial fusion and fission. Mitochondria are easily damaged by various external stimuli. Damaged mitochondria are cleared by autophagy to maintain the stability of mitochondrial dynamics, which is important to ensuring a healthy mitochondrial network. Defects in this machinery cause a range of diseases especially affecting the cardiovascular system (12). Notably, mounting evidence demonstrates that some of the benefits of aerobic exercise against CVD occur through its effects on three underlying mitochondrial aspects: mitochondrial biogenesis, mitochondrial fusion and fission, and mitochondrial autophagy.

## Mitochondrial Biogenesis and CVDs

Mitochondrial biogenesis requires coordination of nuclear and mitochondrial DNA (12). It is critical to healthy mitochondrial dynamics and further affects myocardial function. Mitochondrial biosynthesis is regulated by the peroxisome proliferator–activated receptor gamma coactivator-1 (PGC-1) family. This includes PGC-1α, PGC-1β, and peroxisome proliferator-activated receptor gamma (PPARγ). PGC-1α plays a vital role in regulating cardiac metabolism. Activation of PGC-1 family transcription factors under conditions of cellular energy requirements, such as cell growth, hypoxia, glucose deprivation, or exercise, enhances mitochondrial remodeling or biosynthesis and restores intracellular energy balance (13). PGC-1 knockdown in mice induces cardiomyopathy *via* fragmentation and elongation of cardiac mitochondria, which is related to changes in mitofusin 1 (MFN1), optic atrophy 1 (OPA1), and dynamin-related protein 1 (DRP1) expression (14).

Multiple transcription factors are found downstream of PGC-1α, and peroxisome proliferator–activated receptors (PPARs), nuclear respiratory factor 1 (NRF1), and mitochondrial transcription factor A (TFAM) are all involved in mitochondrial biogenesis (15). PPARα is a member of the nuclear receptor superfamily of PPARs. PPARs participate in decomposition and metabolism of fatty acids and play a key role in maintaining myocardial energy metabolism (16). Specific knockout of PPARα results in cardiac hypertrophy and fat accumulation in rats, and ultimately heart failure leads to animal death (17, 18). In addition, PPARα can ameliorate cardiac hypertrophy caused by hypertension. The PPARα/NRF2 signaling pathway protects the heart from remodeling induced by stress overload, and up-regulation of PPARα protein expression can improve cardiac hypertrophy (19). NRF1/2 is a key component in regulating nuclear coding of mitochondrial proteins and is closely involved in mitochondrial biogenesis. In models of cardiac hypertrophy and heart failure, gene expression and protein levels of NRF1 and TFAM and protein levels of NRF2 are decreased in the cardiac tissue (20).

## Aerobic Exercise Improves CVDs by Regulating Mitochondrial Biogenesis

Mitochondrial biogenesis is inhibited in CVDs (21). The number and ATP production of myocardial mitochondria and mitochondrial synthesis regulatory factors increased in rats after an 8-week treadmill test, indicating that aerobic exercise could improve myocardial energy supply and cardiac function by promoting biosynthesis of myocardial mitochondria (22). Treadmill exercise helps improve CVDs; thus, aerobic exercise may exert direct benefit on mitochondrial biogenesis.

In line with this, aerobic exercise can activate the silencing regulatory protein 3 (SIRT3)/PGC-1α/phosphatidylinositol 3 kinase (PI3K)/Akt signaling pathway, resulting in improved mitochondrial biogenesis (23). Such physical activity can also activate the AMPK/PGC-1α pathway in myocardial tissue. PGC-1α can upregulate expression of mitofusin2 (MFN2) protein, which promotes mitochondrial biogenesis and mitochondrial fusion (13). Further, aerobic exercise enhances protein levels of PGC-1α and NRF2, which increases the mRNA levels of *TFAM* and *NRF1* and increases mitochondrial DNA replication. NRF2 promotes expression of various antioxidant enzymes, thus reducing the oxidative stress level of myocardial tissue (24). Thus, aerobic exercise can promote expression of PGC-1α, NRF1, and NRF2, and expression of regulatory factors and induction of mitochondrial biogenesis improve CVDs.

## Myocardial Mitochondrial Fusion/Fission and CVDs

Mitochondria constantly undergo fission and fusion. After mitochondrial fission, offspring mitochondria with higher membrane potential enter the next round of fusion to maintain mitochondrial number and function. These processes are regulated by mitochondrial fusion and fission proteins, and are particularly important for maintaining normal mitochondrial function (25). Mitochondrial fusion proteins include MFN1, MFN2, and OPA1. MFN1 and MFN2 mediate fusion of the outer mitochondrial membrane (OMM), while OPA1 mediates fusion of the inner mitochondrial membrane. DRP1 serves as a critical effector of mitochondrial fission (26).

In CVDs, mitochondrial morphology and fusion-related proteins exhibit alterations. For example, severe mitochondrial fragmentation occurs in myocardial ischemia–reperfusion injury (I/R) models, which significantly inhibits OPA1 expression (27). Further, there is an abnormal increase in myocardial mitochondria, downregulation of mitochondrial biogenesis-related genes, and aggravation of cardiomyopathy after specific deletion of *Mfn1* and *Mfn2* in the mouse myocardium (28). Mitochondrial fission was caused by translocation of phosphorylated DRP1 from the cytoplasm to the mitochondria (26). The use of DRP1 inhibitor reduced DRP1 translocation to the mitochondria, improved the structure and function of the mitochondria, and alleviated myocardial hypertrophy and myocardial fibrosis (29, 30). Besides the positive changes in mitochondrial dynamics in cardiovascular diseases, inhibiting mitochondrial fission may also lead to heart impairment (31, 32). Therefore, both mitochondrial fusion and fission are essential for CVD development. A balanced state of mitochondrial fission and fusion are conducive to disease prevention and improved prognosis.

## Aerobic Exercise Improves CVDs by Regulating Mitochondrial Fusion and Fission

In CVDs, mitochondria exhibit a high rate of fission and a low rate of fusion, resulting in imbalanced mitochondrial dynamics (12). Notably, aerobic exercise can increase expression of PGC-1α mRNA and protein to improve the level of MFN2 protein and promote mitochondrial fusion (33). Swimming training can downregulate the mir-30B-p53-DRP1 pathway, reduce the contents of p53 and DRP1 proteins in the mouse myocardium, and inhibit myocardial mitochondrial fission (34). The swimming training lasted 8 weeks, 5 days/week. At the beginning, the swimming duration is 30 min. The swimming time was increased by 10 min ever week. Therefore, swimming duration maintained at 90 min after 7–8 weeks. Then, the mice trained twice a day with an interval of 6 h. Aerobic exercise training can induce increased mitochondrial fusion (upregulating the expression of MFN2 and OPA1 proteins) and decreased mitochondrial fission (downregulating the expression of DRP1 protein), and promote mitochondrial kinetic remodeling, effectively alleviating mitochondrial dysfunction in rats with myocardial infarction (MI) (35). After moderate aerobic exercise training, the mRNA levels of *Ppargc1*α, *Opa1, Mfn2*, and *Drp1* significantly increased and the diastolic parameters improved in spontaneously hypertensive rats (SHRs) (22). These results suggest that aerobic exercise maintains the balance of mitochondrial fusion and fission in the SHR myocardium and improves mitochondrial function. A comparison of the myocardial mitochondria of animals with heart failure who exercised vs. did not exercise revealed that the number of myocardial mitochondria in the exercise group was decreased and mitochondrial size was increased. Further experiments showed that exercise increased MFN1, MFN2, and DNM1L GTPase activity in the myocardium and reversed the translocation of DNM1L to the mitochondria (8). Therefore, aerobic exercise may have protective effects on the balance of mitochondrial fusion and fission in CVDs.

## Myocardial Mitochondrial Autophagy and CVDs

Mitochondrial autophagy is a selective form of autophagy, which is specialized to remove aging or irreversibly damaged mitochondria and plays a decisive role in control of mitochondrial quality (36). Mitochondrial autophagy and fission can coordinate with each other, and mitochondrial autophagy can remove damaged mitochondrial parts formed by mitochondrial fission and keep mitochondria healthy (37). DRP1 migration and mitochondrial autophagy activation occur almost simultaneously after transverse aortic constriction (TAC) treatment, indicating that mitochondrial autophagy is closely related to mitochondrial fission (38).

Mitochondrial autophagy may occur through Parkin-dependent or Parkin-independent mechanisms (12). In Parkin-mediated mitotic phagocytosis, PTEN-induced putative kinase 1 (PINK1) recruits Parkin to the outer membrane of mitochondria. Once recruited to the mitochondria, Parkin ubiquitinates several mitochondrial outer membrane proteins, including the mitochondrial fusion proteins MFN1 and MFN2, Miro, Translocase of OMM 20, and voltage-dependent anion channel. Then, the selective autophagy adapter protein P62/Sequestosome 1 is recruited to the mitochondria to interact with LC3 and initiate mitochondrial autophagy (36, 37, 39). Meanwhile, ubiquitination and proteasome degradation of MFN1 and MFN2 results in mitochondrial fission and fragmentation, which further induces mitochondrial autophagy (40). PINK1 and Parkin protein levels are significantly reduced in heart failure models (41). PINK1 knockout mice have a significantly increased number of atherosclerotic lesions (42). Also, Parkin knockout mice show excessive myocardial hypertrophy, myocardial fibrosis, and left ventricular systolic dysfunction in response to TAC (43).

Parkin-independent mitochondrial autophagy occurs independently of Parkin; some autophagy receptor proteins are located in mitochondria and interact with LC3 to recruit autophagosomes to damaged mitochondria. These autophagy receptor proteins include BCL-2/adenovirus E1B19-kDa interacting protein 3 (BNIP3), NIX (also called BNIP3L), and Fun14 Domain–containing 1 (FUNDC1) (36, 44, 45). Lowering of BNIP3 protein levels impairs mitochondrial function, which in turn leads to impaired myocardial cell function (46); also, *FUNDC1* gene knockout exacerbates I/R injury (47).

Mitochondrial autophagy has a significant protective effect on the heart, and autophagy activation is observed in the boundary region of the subacute stage of MI (1 week after MI) (48). Enhancement of autophagy with rapamycin 2 weeks after coronary artery ligation ameliorates cardiac dysfunction and maladaptive remodeling, whereas inhibition of autophagy by buffamycin A1 worsens cardiac dysfunction (49). In conclusion, insufficient mitochondrial autophagy is closely related to CVD development.

## Aerobic Exercise Improves CVDs by Regulating Mitochondrial Autophagy

CVD pathology is associated with weakening of mitochondrial autophagy. Yet, 8 weeks of aerobic exercise on a treadmill can increase the LC3II/LC3I ratio, and thus upregulate Beclin1, LC3, and BNIP3 in the rat myocardium to promote myocardial autophagy (50). For treadmill excise study, rats were trained on a treadmill with an exercise intensity and schedule set at 70% of maximum aerobic capacity. The training group run for 60 min/day, 5 days per week at 10 m/min speed for 8 weeks. After the treadmill excise training, PINK1, Parkin, ubiquitin, P62, and LC3 levels were significantly increased in the rat skeletal muscle (51). Consistent with this finding, exercise improves the oxidative capacity of myocardial mitochondria in animals with heart failure, and this improvement in oxidative capacity is related to reconstruction of autophagy flux. Exercise stimulates mitochondrial autophagy flux by increasing Parkin recruitment in myocardial mitochondria (8). Notably, aerobic exercise training enhances the PINK1/Parkin signaling pathway, thus inducing mitochondrial autophagy (52). This physical activity also upregulates SIRT3 expression, enhances the antioxidant capacity of the body, improves the quality of mitochondria, and helps alleviate cardiac dysfunction in mice after MI (53). These studies suggested that aerobic exercise induces mitochondrial autophagy and improves CVDs by increasing the expression of autophagy-related and mitochondrial autophagy–related proteins.

## Conclusions and Perspectives

Abundant evidence illustrates the key role of mitochondrial dynamics in cardiac homeostasis. Imbalanced mitochondrial fusion and fission, insufficient mitochondrial autophagy, and weakened mitochondrial biogenesis are the pathological factors leading to CVD occurrence and development. Regular aerobic exercise is a simple and effective way to balance mitochondrial dynamics and improve mitochondrial function, which can build human health. MFN2 and OPA1, which are beneficial to mitochondrial fusion and biogenesis, can be elevated by aerobic exercise. DRP1-induced mitochondrial fission can be inhibited by aerobic exercise in cardiovascular diseases. PINK1/parkin signaling is still the main targeting pathway that induces mitochondrial autophagy following aerobic exercise in cardiovascular diseases. However, one study indicated that acute exercise had no effect on the proteins involved in mitochondrial dynamics. Indeed, after exercise training, the proteins involved in mitochondrial dynamics changed significantly in normotensive control rats, but remained unchanged in spontaneously hypertensive rats (22). This finding aligned with another study finding that myocardial mitochondrial dynamics-related proteins did not change after acute exercise in rats (54).

Aerobic exercise can prevent or alleviate CVD development by regulating proteins involved in mitochondrial dynamics. Exercise can reduce mitochondrial fission, enhance mitochondrial fusion and improve mitochondrial autophagy or biogenesis. Regarding to the type of exercise, we compared some excise programs of treadmill training with swimming, and also summarized the influence of different exercise programs on mitochondrial dynamics (Table 1). Treadmill training seems to have a greater effect on modulation of the cardiac proteome than swimming (55). While we can control the intensity and duration of treadmill training to calculate the amount of exercise, swimming training is more difficult to control. Some animals do not exhibit continuous swimming behavior, but rather tend to dive or swing, which results in intermittent hypoxia and may affect interpretation of the results.

**Table 1:** Comparison of different exercise training programs.

	**Treadmill**				**Swimming**
Time	60 min/day	10 or 60 min/day	20–60 min/day	60 min/day	20–60 min/day
Frequency	5 days/week	6 days/week	Every day	Every day	5 days/week
Period	8 weeks	8 weeks	11 weeks	4 weeks	8 weeks
Protocol	Ran on a treadmill at 60–70% VO_2_max intensity for 60 minutes.	In the first 4 weeks, 10 m/min, 0% slope, 10 min/day. Then 20 m/min, 5% slope, 60 min/day for 4 weeks.	The speed and incline of the treadmill gradually increased. Starting with low workloads (25 min, 35% Vmax and 0% gradient), to the end of high workloads (60 min, 70%Vmax and 25% gradient).	The rats initially ran 30 min daily at a speed of 10 m/min and gradually increased by 10 min in duration and 2 m/min in speed each day until reaching 60 min per day at a speed of 16 m/min.	Training time was increased by 10 min per week.
Influence on mitochondrial dynamics	Promoted fusion and inhibited fission (expression of MFN2, PGC-1α and OPA1 increased, expression of DRP1 decreased).	Promoted biogenesis (expression of PGC-1α and NRF2 increased).	Promoted autophagy (expression of LC3II and P62 increased).	Promoted biogenesis (SIRT1/PGC-1α signaling pathway was activated).	Promoted fusion and inhibited fission (expression of MFN1, MFN2, and OPA1 increased, expression of DRP1 decreased).

Mitochondrial dynamics, including mitochondrial biogenesis, mitochondrial fusion or fission, and mitochondrial autophagy, can be used as disease prevention and treatment targets under exercise intervention. Moreover, the balance between mitochondrial dynamics may be a key factor in disease occurrence or development. However, the mechanism of aerobic exercise intervention in mitochondrial dynamics is complex and needs further long-term research due to its gradual effects and individual differences. Improper exercise can also lead to arthritis, decreased immunity, etc. Therefore, verified and reasonable individualized exercise prescription has important guiding significance for health. It is important to carry out long-term and proper exercise intervention.

The concept of aerobic exercise for disease prevention is increasingly popular, while the number of subhealthy people and older adults is increasing every year. Reasonable exercise prescription intervention for these populations or people with a tendency for organ disease development can significantly reduce disease occurrence. Further studies are needed to explore this idea.

## Author Contributions

Y-lW: conceptualization and methodology. CG: data curation and writing—original draft preparation. JY: writing—reviewing and editing. LZ: visualization and investigation. GW: software. All authors contributed to the article and approved the submitted version.

## Conflict of Interest

The authors declare that the research was conducted in the absence of any commercial or financial relationships that could be construed as a potential conflict of interest.

## Publisher's Note

All claims expressed in this article are solely those of the authors and do not necessarily represent those of their affiliated organizations, or those of the publisher, the editors and the reviewers. Any product that may be evaluated in this article, or claim that may be made by its manufacturer, is not guaranteed or endorsed by the publisher.
